# CT radiation dose reduction with tin filter for localisation/characterisation level image quality in PET-CT: a phantom study

**DOI:** 10.1186/s40658-024-00703-6

**Published:** 2024-11-25

**Authors:** Natalie Anne Bebbington, Lone Lange Østergård, Kenneth Boye Christensen, Paw Christian Holdgaard

**Affiliations:** 1 Siemens Healthcare A/S, Borupvang 9, Ballerup, 2750 Denmark; 2 Department of Nuclear Medicine, Lillebaelt University Hospital, Beriderbakken 4, Vejle, 7100 Denmark

**Keywords:** CT, PET, Tin filter, Spectral shaping, Dose reduction, Hybrid imaging

## Abstract

**Background:**

The tin filter has allowed radiation dose reduction in some standalone diagnostic computed tomography (CT) applications. Yet, ‘low-dose’ CT scans are commonly used in positron emission tomography (PET)-CT for lesion localisation/characterisation (L/C), with higher noise tolerated. Thus, dose reductions permissible with the tin filter at this image quality level may differ. The aim was to determine the level of CT dose reduction permitted with the tin filter in PET-CT, for comparable image quality to the clinical reference standard (CRS) L/C CT images acquired with standard filtration.

**Materials and methods:**

A whole-body CT phantom was scanned with standard filtration in CRS protocols, using 120 kV with 20mAs-ref for bone L/C (used in ^18^F-Sodium Fluoride (NaF) PET-CT) and 40mAs-ref for soft tissue L/C (used in ^18^F-Fluorodeoxyglucose (FDG) PET-CT), followed by tin filter scans at 100 kV (Sn100kV) and 140 kV (Sn140kV) with a range of mAs settings. For each scan, effective dose (ED) in an equivalent-sized patient was calculated, and image quality determined in 5 different tissues through quantitative (contrast-to-noise ratio) and qualitative (visual) analyses. The relative dose reductions which could be achieved with the tin filter for comparable image quality to CRS images were calculated.

**Results:**

Quantitative analysis demonstrated dose savings of 50–76% in bone, 27–51% in lung and 8–61% in soft tissue with use of the tin filter at Sn100kV. Qualitative analysis demonstrated dose reductions using Sn100kV in general agreement with the dose reductions indicated by quantitative analysis. Overall, CT dose reductions of around 85% were indicated for NaF bone PET-CT, allowing whole-body CT at just 0.2mSv ED, and a 30–40% CT dose reduction for FDG PET-CT using Sn100kV (1.7-2.0mSv), providing comparable image quality to current CRS images with standard filtration. Sn140kV demonstrated limited value in CT dose reduction.

**Conclusions:**

Large CT dose reductions can be made using the tin filter at Sn100kV, when imaging bone, lung and soft tissue at L/C level CT image quality in PET-CT. As well as reducing the risk of inducing a cancer in later life, such dose reductions may also impact PET-CT practice, such as justifying cross-sectional over planar imaging or justifying PET-CT in younger patients.

**Supplementary Information:**

The online version contains supplementary material available at 10.1186/s40658-024-00703-6.

## Background

Hybrid positron emission tomography (PET)-computed tomography (CT) imaging is widely used for diagnosis, staging and monitoring of disease progression in a wide range of malignancies. The PET scan provides images at the molecular level, representing metabolic tumour activity, and is highly sensitive for detecting metastases. For that reason, with oncological indications a whole-body (‘eyes to thighs’) scan range is usually performed as a minimum. A CT scan is also usually performed in addition, commonly at the localisation and characterisation (L/C) level [1–5], such that foci of increased PET tracer uptake can be accurately located within the body and the disease aetiology characterised through the morphological information provided. In addition, the CT images are used for attenuation correction (AC) of the PET signal: a process through which the PET images are compensated for image counts lost through attenuation at greater depths within the body and through higher density tissues [6].

The practice of performing CT for L/C and AC in PET-CT is referred to as ‘low-dose CT’, since exposure settings are typically lower than those used in standalone ‘fully-diagnostic’ CT. However, since a whole-body scan range is typically used for oncology indications, and the comparator, namely fully-diagnostic CT, is a high-dose technique, the CT radiation doses reported for L/C level CT in PET-CT are actually not low [1–4]. On the contrary, national dose surveys show that the dose for L/C level CT in fact usually matches or exceeds the dose from the PET scan, with doses of around 5 mSv typically delivered each for the Fluorine-18 Fluorodeoxyglucose (^18^F-FDG) tracer and the L/C CT scan [2, 6]. This gives a combined dose of at least 10 mSv, and an associated risk of inducing a fatal cancer in later life of 1 in 2000, using the widely accepted assumption of 5% per Sievert of radiation delivered [7]. Since 2.2 million PET-CT scans are performed in the United States (US) each year based on data from 2019 to 2020 [8], at current PET-CT doses around 1100 fatal cancers would be induced per year in the US alone.

In recent years, a tin (Sn) filter has become available on newer generation Siemens Healthineers standalone CT, PET-CT and single photon emission computed tomography (SPECT)-CT systems, in addition to the standard aluminium hardware filter. The tin filter, in addition to its role in increasing spectral separation in dual-energy CT [9], can also be used for CT dose optimisation in single-energy CT, since it filters out a greater proportion of the lower energy photons in the X-ray beam, which are otherwise absorbed by the patient. This spectral shaping technique with use of the tin filter has demonstrated large dose-savings for imaging of pathologies at the image quality level required in standalone CT, typically when imaging high contrast abnormalities without use of contrast media, for example in bone [10–12], lung [13–15], kidney stones [16, 17] and cardiovascular calcifications [18, 19]. However, there are no publications as yet demonstrating by how much the CT radiation dose can be reduced at the L/C CT image quality level, commonly used in hybrid imaging.

The aim of this study was to evaluate the level of dose-saving which could be made with the tin filter, whilst achieving L/C level CT image quality, obtained in clinical reference standard (CRS) bone and soft tissue PET-CT imaging with standard filtration, through qualitative and quantitative analysis of whole-body phantom data.

## Methods

### Equipment

A PBU 60 whole-body adult patient phantom (Kyoto Kagaku, Kyoto, Japan) was used in standard and obese configurations. In the standard configuration the phantom represents a standard-sized adult male (maximum dimensions x = 21.1 cm, y = 29.2 cm, thorax), whilst in the obese configuration, two additional plates of fat-equivalent density are added to the front and back of the phantom to represent an obese adult male (maximum dimensions x = 33.4 cm, y = 40.0 cm, abdomen). The phantom contains detailed anatomy adequately representing that of patient and is thus a valuable tool for evaluating CT image quality in patients, even though it represents a healthy patient and does not contain structures representing clinical abnormalities.

CT imaging was performed using a Biograph Vision 600 PET system with 128-slice SOMATOM Definition Edge CT (Siemens Healthineers, Knoxville, Tennessee, United States), equipped with standard aluminium and additional tin hardware filters.

### Image acquisition and reconstruction

The phantom was scanned in standard and obese configurations with arms up from vertex to knee. Two CRS scans were made with standard filtration for each phantom configuration: firstly using the standard clinical protocol for ^18^F Sodium-fluoride (NaF) PET-CT in this department, with a tube voltage of 120 kV and effective reference mAs (mAs-ref) of 20 with tube current modulation applied. The term ‘effective’ represents that the pitch factor is considered in the reported mAs value, whilst ‘reference’ represents the mAs which would be used in a standard sized (70–80 kg) patient [20]. The second CRS acquisition was made with 120 kV and 40 mAs-ref, representing the CRS protocol for ^18^F-FDG PET-CT, optimised predominantly for soft tissue visualisation.

Multiple tin filter scans were then acquired for each phantom configuration, at the two tube voltage settings at which the tin filter can be applied on this system, 100 kV (Sn100kV) and 140 kV (Sn140kV), with a range of mAs-ref settings as detailed in Table 1, along with other relevant acquisition settings.

When using the tin filter, the reference mAs is to be increased somewhat if aiming to deliver a comparable dose to the patient as the standard filtration protocol, since such a large proportion of the X-rays are removed from the beam, including removal of X-rays both at desired and undesired energies. Differences in pitch and rotation time between acquisitions, as shown in Table 1, were simply to allow the system to deliver a higher or lower effective mAs as required, with the respect to the maximum and minimum deliverable tube current (mA) on the system and would otherwise have negligible impact on image quality and dose.

For all scans, CARE kV was operated in ‘semi’ mode, meaning that the tube voltage was fixed. Dose saving was optimised for bone (sliding bar value 5), since this department was primarily interested in implementation of the tin filter for ^18^F-NaF bone PET-CT. Also for this reason, the ‘spine’ organ characteristic was used for tube current modulation.

**Table 1:** Overview of CT acquisition settings used

Scan protocol number	Tin filter	Tube voltage (kV)	mAs (mAs-ref)	Organ characteristic	CARE kV mode	CARE kV exam type	Rotation time (s)	Pitch	Collimation
1	Not applied	120	20	Spine	Semi	Bone	0.5	1.5	128 * 0.6 mm
2	Not applied	120	40	Spine	Semi	Bone	0.5	1.5	128 * 0.6 mm
3	Applied	Sn140	100	Spine	Semi	Bone	0.5	0.8	128 * 0.6 mm
4	Applied	Sn140	50	Spine	Semi	Bone	0.5	1.5	128 * 0.6 mm
5	Applied	Sn140	25	Spine	Semi	Bone	0.5	1.5	128 * 0.6 mm
6	Applied	Sn140	12	Spine	Semi	Bone	0.5	1.5	128 * 0.6 mm
7	Applied	Sn140	7	Spine	Semi	Bone	0.5	1.5	128 * 0.6 mm
8	Applied	Sn100	400	Spine	Semi	Bone	1.0	0.5	128 * 0.6 mm
9	Applied	Sn100	350	Spine	Semi	Bone	1.0	0.5	128 * 0.6 mm
10	Applied	Sn100	300	Spine	Semi	Bone	1.0	0.8	128 * 0.6 mm
11	Applied	Sn100	250	Spine	Semi	Bone	1.0	0.8	128 * 0.6 mm
12	Applied	Sn100	200	Spine	Semi	Bone	0.5	0.8	128 * 0.6 mm
13	Applied	Sn100	150	Spine	Semi	Bone	0.5	0.8	128 * 0.6 mm
14	Applied	Sn100	100	Spine	Semi	Bone	0.5	0.8	128 * 0.6 mm
15	Applied	Sn100	50	Spine	Semi	Bone	0.5	1.5	128 * 0.6 mm
16	Applied	Sn100	25	Spine	Semi	Bone	0.5	1.5	128 * 0.6 mm

mAs-ref = reference mAs; Sn100 = tin filter applied to X-rays generated using a peak tube voltage of 100 kV; Sn140kV = tin filter applied to X-rays generated using a peak tube voltage of 140 kV

Reconstructions were made for all scans with the third generation Advanced Modeled Iterative Reconstruction (ADMIRE) strength 3, in 3 mm slice thickness with 1 mm overlap (2 mm increment), with kernels Br38 (regular body kernel for soft tissue visualisation) and Br51 (regular body kernel with low-dose CT for bone visualisation).

### Dose calculation

For each scan, dose length product (DLP, mGy.cm) was recorded from the Patient Protocol file. Effective doses (ED, mSv) were derived using the 0.013 DLP to ED conversion factor for whole-body CT [21]. Despite using phantoms of different sizes, size-specific dose estimates (SSDEs) were not made, since the conversion factors published in the American Association of Physicists in Medicine (AAPM) Report 204 are reasonably applicable only to thoracic, abdominal and pelvic scan ranges [22], and are not available for the whole-body scan range.

### Quantitative analysis

**Fig. 1:**
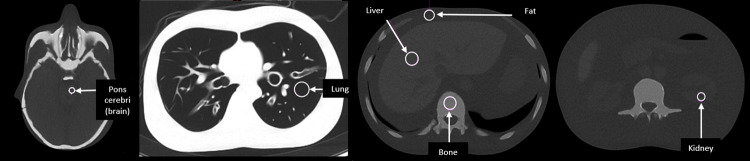
ROI placement in homogenous areas of the brain, lung, bone, fat, liver and kidney

Reconstructions made with the Br51 kernel were used for quantitative analysis in *syngo*.via software version VB70A (Siemens Healthineers, Erlangen, Germany). 6 regions of interest (ROIs) of area 0.64 to 2.51 cm^2^ were placed on homogenous areas of the phantom in the pons cerebri (brain), lung, spine (bone), fat, liver and kidney (Fig. 1). The ROIs we placed firstly on the 120 kV/40 mAs CRS scan, and then copied to all other reconstructions. Mean and standard deviation (SD) Hounsfield unit (HU) values were recorded for each ROI in each dataset. Tissue contrast of the brain, lung, bone, liver and kidney were calculated relative to fat, by calculating the difference in HU between the tissue of interest and fat. SD of HU values in each ROI were used to determine noise, and the contrast-to-noise ratio (CNR) was calculated in each tissue relative to fat thereafter. CNR was calculated using the equation [(*HU*_*tissue*_)-(*HU*_*fat*_)]/(*SD*_*tissue*_), except in the case of lung, in which the equation [(*HU*_*fat*_)-(*HU*_*tissue*_)]/(*SD*_*tissue*_) to give a positive CNR value. For each kV setting for each tissue and each phantom configuration, CNR was plotted against ED, and the percentage dose reductions provided by the tin filter for comparable CNRs to those obtained in the CRS images were calculated.

### Qualitative analysis

**Fig. 2:**
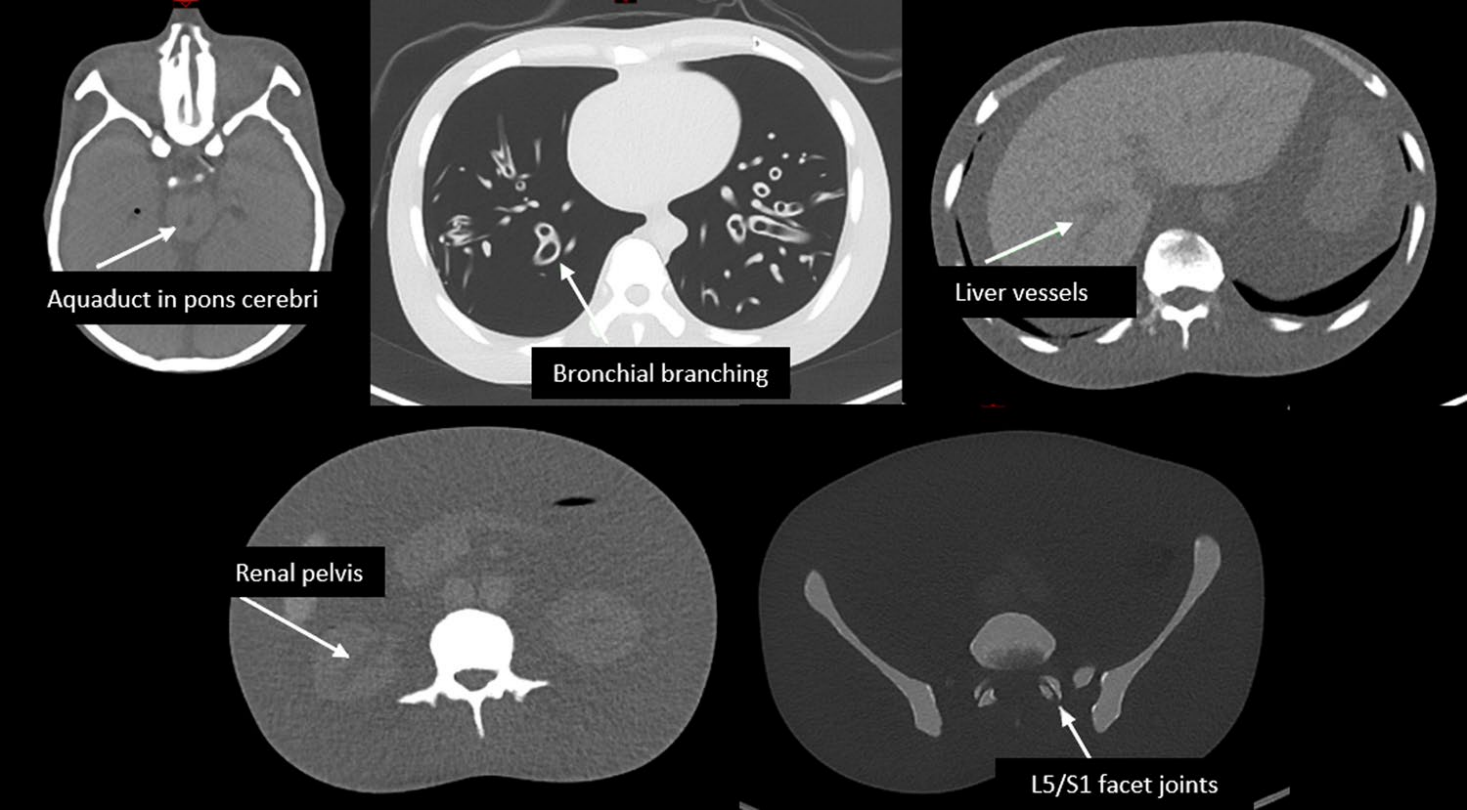
Five anatomical sites used for qualitative image quality assessment. L5 = fifth lumbar vertebra; S1 = first sacral vertebra

Two expert observers, one Consultant Radiologist and one Consultant Nuclear Medicine Physician, qualitatively evaluated the image quality of the reconstructions for each scan at 5 anatomical locations: the aqueduct in the pons cerebri (brain), bronchi (lung), liver vessels (liver), facet joints in the spine between the fifth lumbar and first sacral vertebrae (bone) and the renal pelvis (kidney), as shown in Fig. 2. The Br51 kernel reconstructions were used to evaluate bone and lung, whilst the Br38 reconstructions were used to evaluate the soft tissues. At each anatomical location, an image quality score was assigned by each observer, for each scan, on a scale of 1 to 5: 1 = not detectable, 2 = barely visible (AC only), 3 = acceptable (localisation), 4 = good (characterisation) and 5 = excellent (diagnostic). The image quality scores achieved in each tissue in CRS images provided image quality benchmarks for each observer, and the dose reductions which could be made in each tissue with the tin filter whilst maintaining the image quality benchmark for each respective observer was calculated.

## Results

**Fig. 3:**
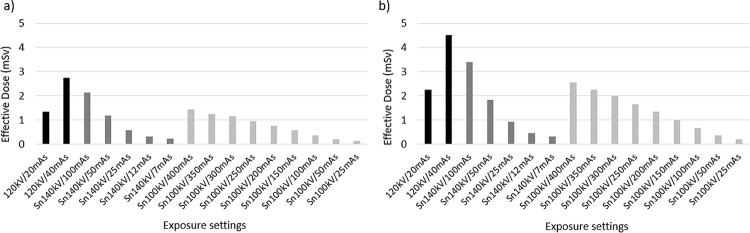
Whole-body ED according to exposure settings for (**a**) standard and (**b**) obese phantom configurations. mAs = milliampere seconds; Sn100kV = tin filter applied to X-rays generated using a peak tube voltage of 100 kV; Sn140kV = tin filter applied to X-rays generated using a peak tube voltage of 140 kV

Figure 3a shows that the 120 kV/20 mAs-ref and 120 kV/40 mAs-ref CRS protocols used by this department for L/C in NaF bone and FDG PET-CT examinations provide whole-body EDs of 1.35 mSv and 2.74 mSv in the standard-sized phantom, respectively. In the obese configuration, these doses increase to 2.25 mSv and 4.51 mSv (Fig. 3b). These figures also demonstrate that when using Sn100kV, a seventeen-to-twenty-fold increase in mAs is required to deliver comparable dose, since such a large proportion of the lower energy photons are removed from the X-ray beam with the tin filter, whilst only a three-to-four-fold difference in mAs is required with Sn140 kV.

**Fig. 4:**
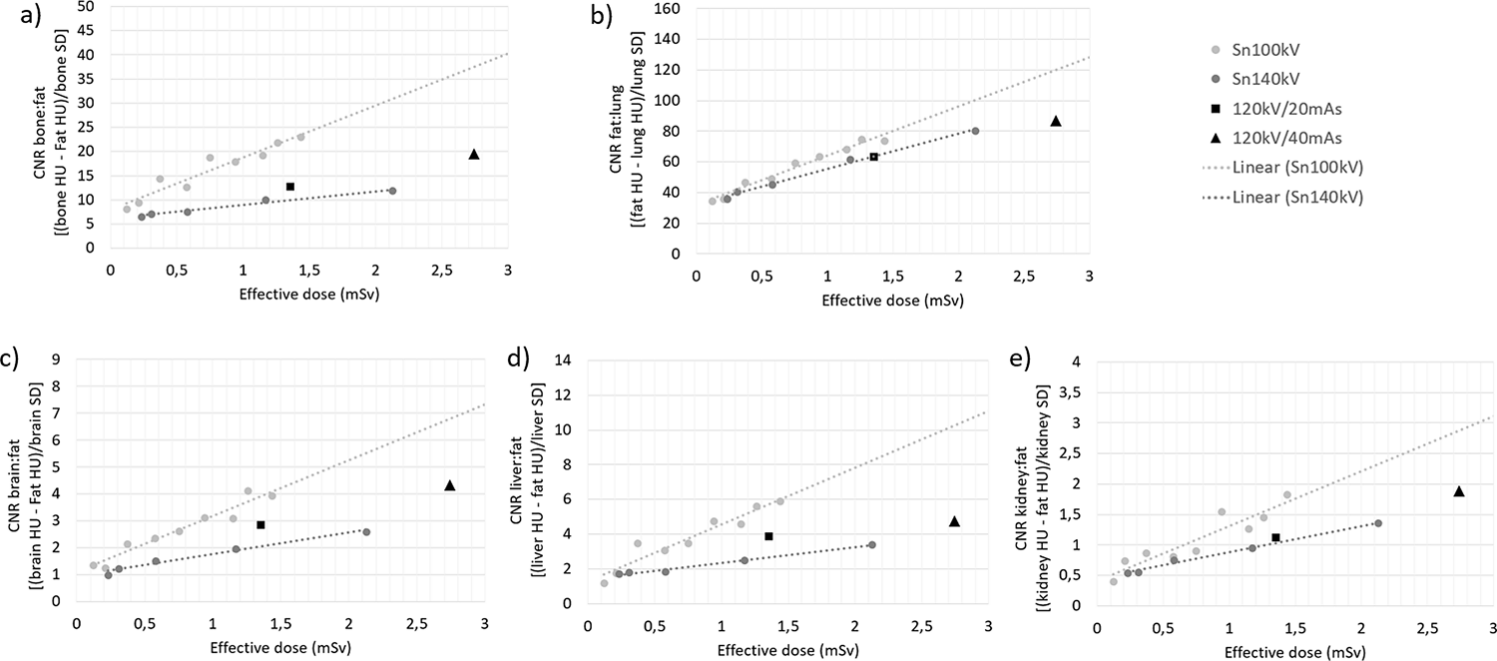
Standard phantom CNR vs. ED for (**a**) bone, (**b**) lung, (**c**) brain, (**d**) liver and (**e**) kidney. CNR = contrast-to-noise ratio; HU = Hounsfield units; mSv = millisievert; SD = standard deviation; mAs = milliampere seconds; Sn100kV = tin filter applied to X-rays generated using a peak tube voltage of 100 kV; Sn140kV = tin filter applied to X-rays generated using a peak tube voltage of 140 kV

Figure 4 demonstrates that for each tissue type, Sn100kV provides improved CNR for comparable dose or allows reduced dose for comparable CNR, in the standard phantom size, at both CRS image quality levels. On the other hand, with use of Sn140kV, lung is the only tissue in which an improvement in CNR can be seen for comparable dose to the CRS, or a slightly reduced dose for comparable CNR, and this is only possible when comparing to the 120 kV/40 mAs CRS protocol.

**Fig. 5:**
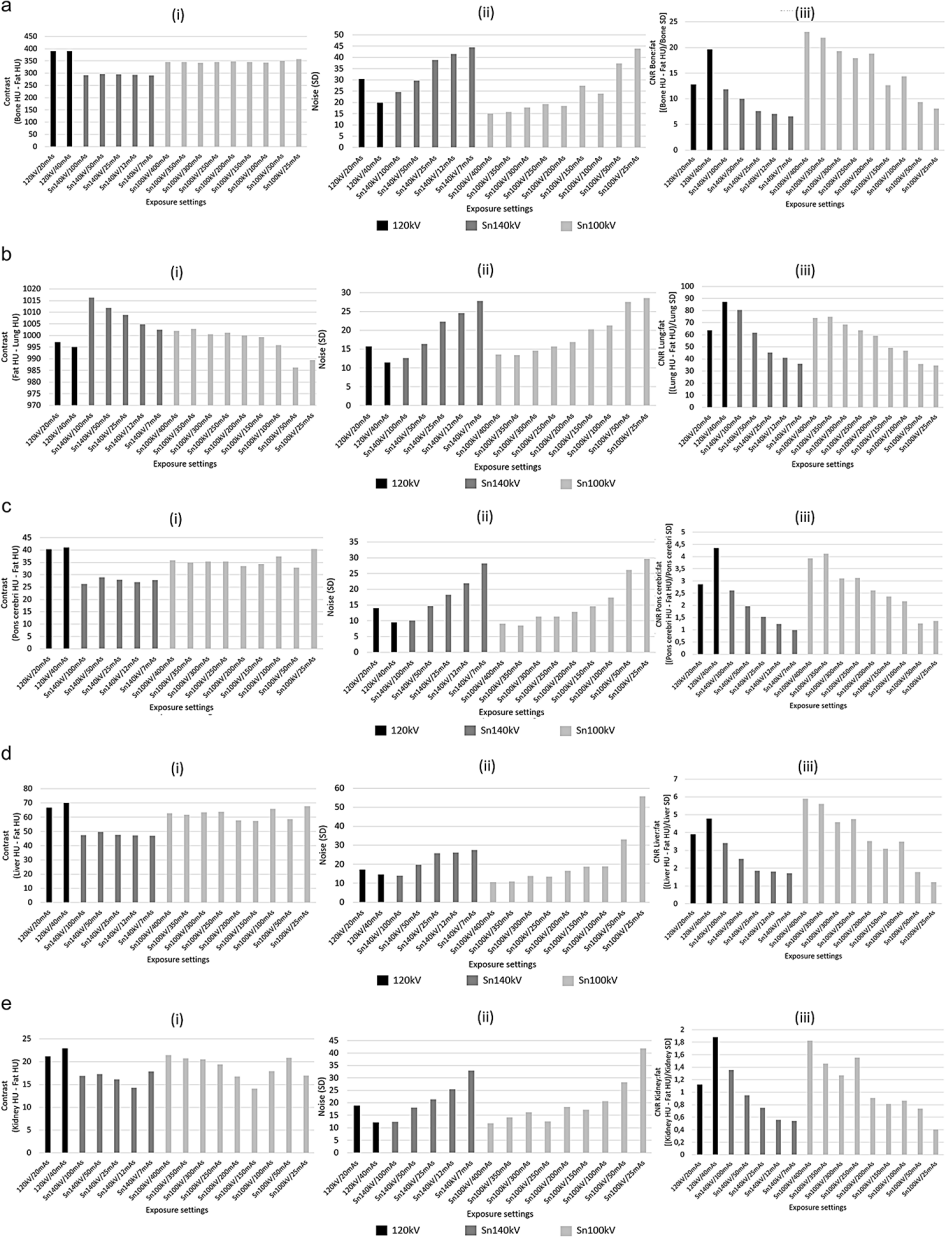
(**a**) Contrast (i), noise (ii) and CNR (iii) values according to exposure setting in bone. (**b**) Contrast (i), noise (ii) and CNR (iii) values according to exposure setting in lung. (**c**) Contrast (i), noise (ii) and CNR (iii) values according to exposure setting in brain. (**d**) Contrast (i), noise (ii) and CNR (iii) values according to exposure setting in liver. (**e**) Contrast (i), noise (ii) and CNR (iii) values according to exposure setting in kidney. HU = Hounsfield units; SD = standard deviation; mAs = milliampere seconds; Sn100kV = tin filter applied to X-rays generated using a peak tube voltage of 100 kV; Sn140kV = tin filter applied to X-rays generated using a peak tube voltage of 140 kV

The contrast, noise and CNR values according to exposure setting on which these CNR data from Fig. 4 are based, are shown in Fig. 5a-e. These data in combination with the dose data from Fig. 3 demonstrate that, whilst image contrast is slightly reduced at Sn100kV as compared with the CRS 120 kV protocol in all tissues except lung, noise is more greatly reduced at the same dose. Hence there is a large overall improvement in CNR at comparable dose, or dose can be reduced for comparable CNR. At Sn140kV on the other hand, image contrast is more greatly reduced than with Sn100kV, and doses are higher for comparable noise, thus CNR is worse at comparable dose. For lung tissue, image contrast improved with increasing average beam energy, hence why a small dose saving could be made at Sn140kV as compared with the 120 kV/40 mAs CRS protocol.

**Fig. 6:**
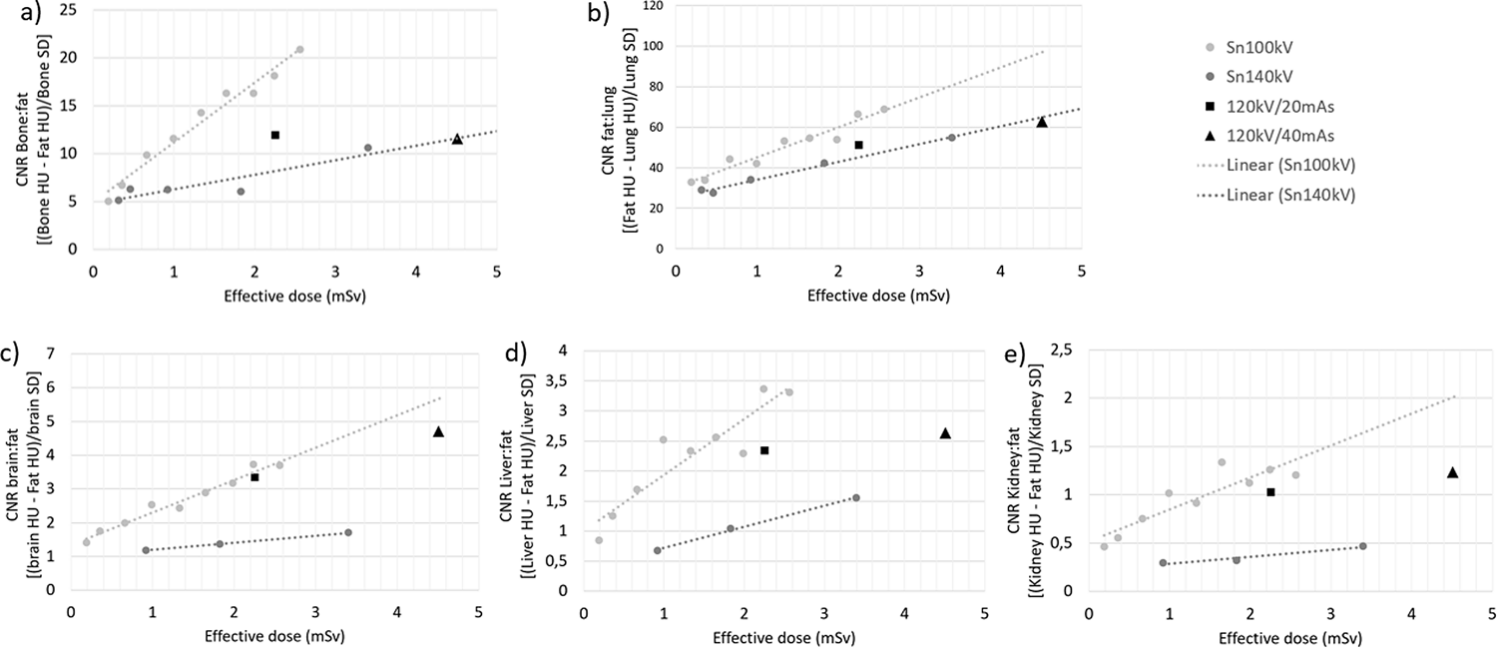
Plots of CNR vs. ED at each tube voltage in the obese phantom for (**a**) bone, (**b**) lung, (**c**) brain, (**d**) liver and (**e**) kidney. CNR = contrast-to-noise ratio; HU = Hounsfield units; SD = standard deviation; mAs = milliampere seconds; mSv = millisievert; Sn100kV = tin filter applied to X-rays generated using a peak tube voltage of 100 kV; Sn140kV = tin filter applied to X-rays generated using a peak tube voltage of 140 kV

The same findings as demonstrated in Fig. 4 for the standard phantom configuration, of large dose savings with Sn100kV, are also made with the obese phantom, as shown in Fig. 6. The equations for the linear fits for CNR versus ED with Sn100kV are provided in Appendix I of the supplementary data, such that expected dose savings with Sn100kV can be interpolated from current dose or CNR levels delivered in non-tin-filter CT protocols scanned at 120 kV.

**Fig. 7:**
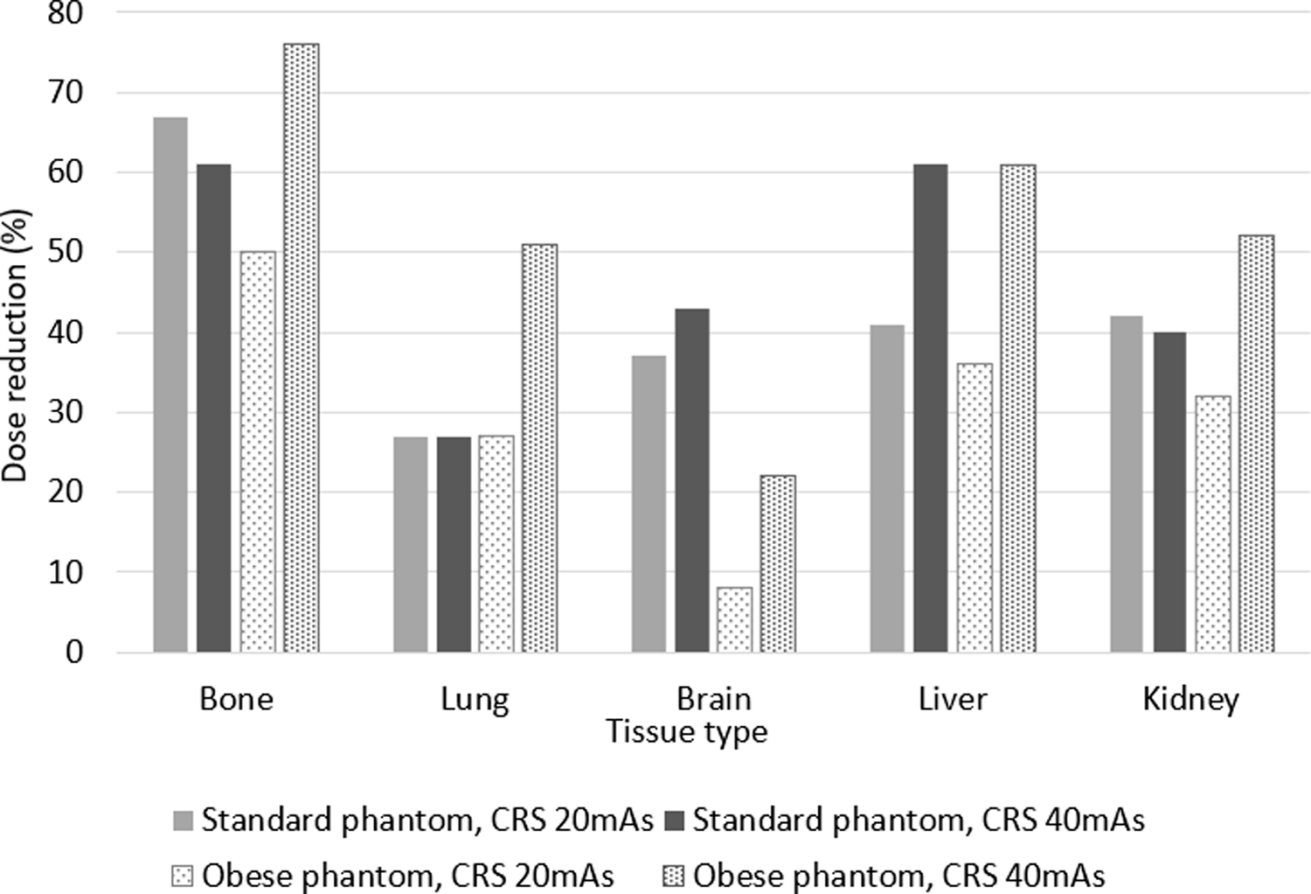
Relative dose-savings for comparable CNR with Sn100kV for the five tissue types, according to phantom configuration and CRS protocol. CRS = clinical reference scan; mAs = milliampere seconds

Figure 7 shows that according to quantitative ED vs. CNR analysis, use of Sn100kV provides dose-reductions for comparable image quality of 50–76% in bone, 27–51% in lung, 36–61% in liver, 32–52% in kidney and 8–43% in the brain. Whilst Sn100kV dose-savings appear greater when comparing to the 120 kV/40 mAs CRS rather than the 120 kV/20 mAs CRS in the obese phantom, no trend was observed in the standard-sized phantom, or between dose savings in standard vs. obese phantom sizes.

**Fig. 8:**
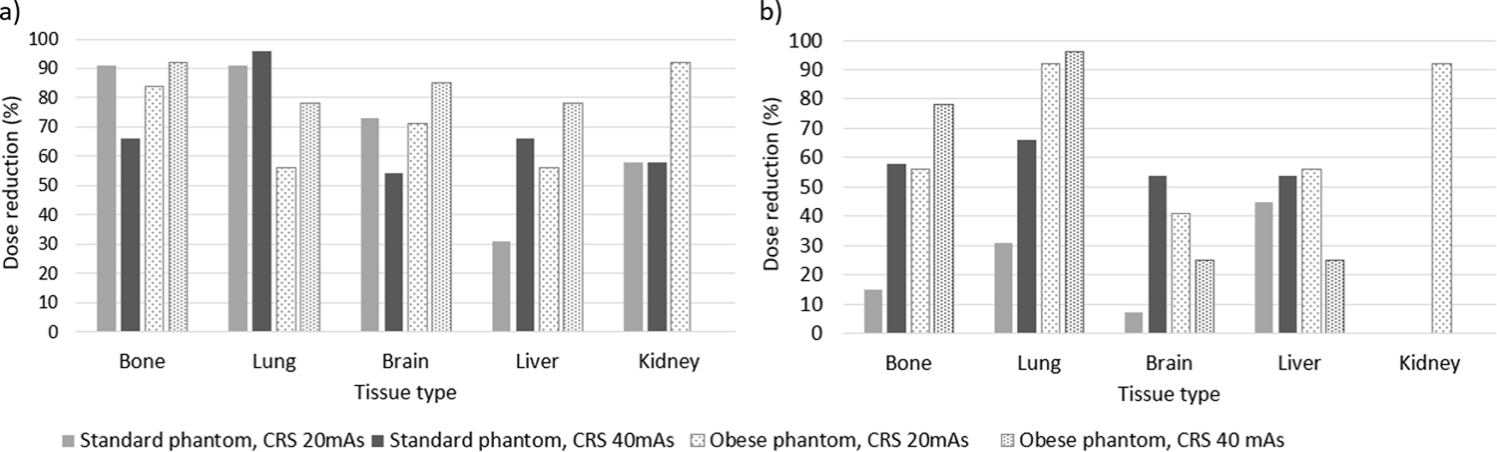
Relative dose-savings with Sn100kV from qualitative evaluation for observers 1 (**a**) and 2 (**b**). CRS = clinical reference scan; mAs = milliampere seconds. Absent bars indicate that 0% dose reduction could be made under that condition

Figure 8 demonstrates that comparable qualitative image quality scores can be achieved with large dose reductions using Sn100kV relative to the CRS protocols at 120 kV, and that the magnitudes of dose-savings suggested by qualitative assessment are in general agreement with those demonstrated by quantitative assessment in Fig. 7.

**Fig. 9:**
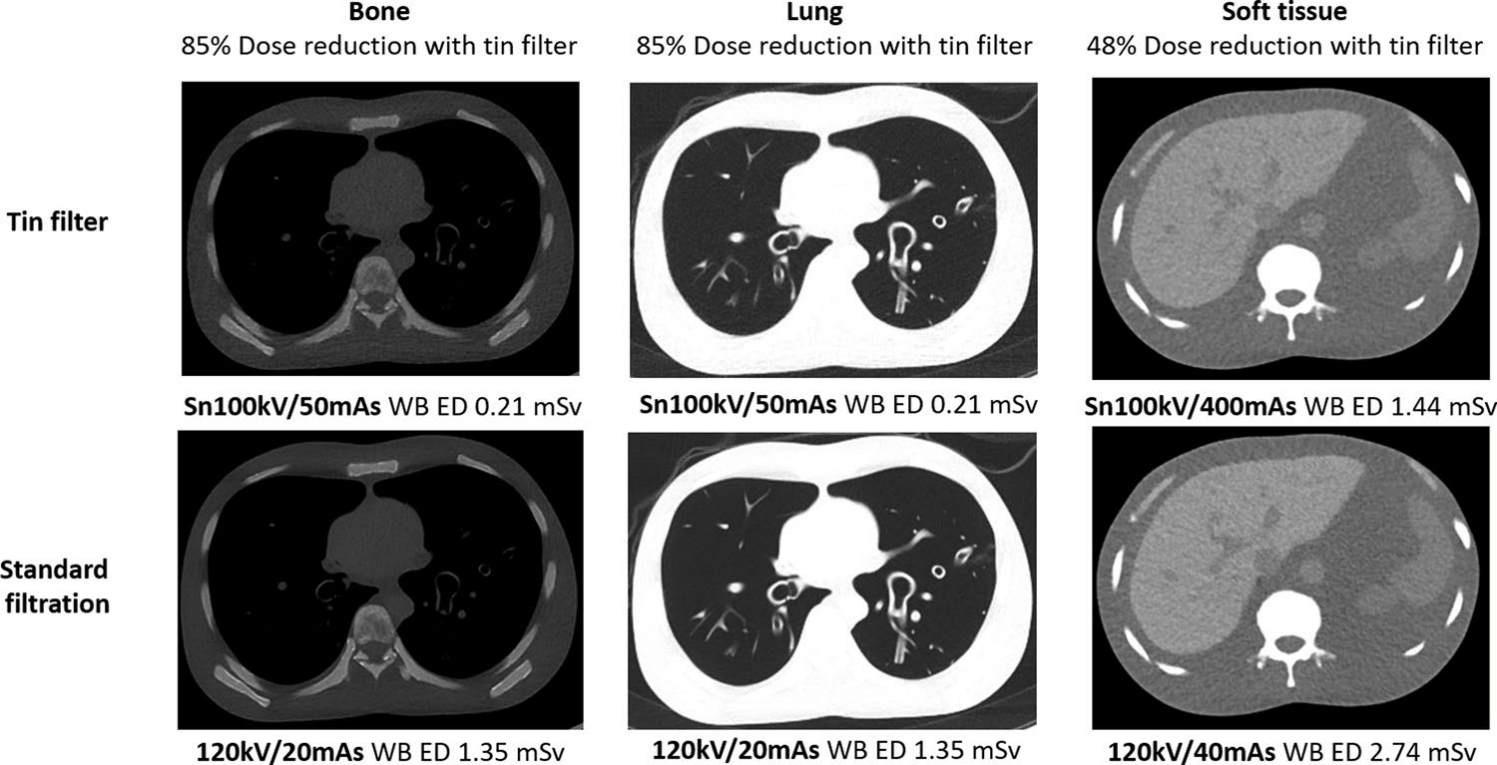
Image quality in bone, lung and soft tissue for CRS and reduced-dose tin filter protocols. ED = effective dose; mAs = milliampere seconds; Sn100kV = tin filter applied to X-rays generated using a peak tube voltage of 100 kV; Sn140kV = tin filter applied to X-rays generated using a peak tube voltage of 140 kV; WB = whole-body

The images from the standard phantom configuration shown in Fig. 9 demonstrate that an 85% dose reduction using Sn100kV/50 mAs-ref (ED 0.2 mSv) provides comparable bone and lung visualisation to the CRS images acquired at 120 kV/20 mAs-ref (ED 1.4 mSv). Figure 9 also demonstrates sufficiently comparable soft tissue image quality for the Sn100kV/400 mAs-ref scan (1.4mSv), for a 48% dose reduction to the CRS scan using 120 kV/40 mAs-ref (ED 2.7 mSv).

## Discussion

### Summary of findings

This study has demonstrated that, despite a greater reference mAs being required with use of tin filter compared with the standard filtration CRS protocol (Fig. 3), Sn100kV allows large dose savings, in standard (Fig. 4) and obese (Fig. 6) phantom sizes. Dose-savings with use of Sn100kV indicated through quantitative (Fig. 7) and qualitative (Fig. 8) assessment were in general agreement, each suggesting that large dose reductions can be made with the tin filter, and indicated that dose reductions of around 85% may be appropriate for bone imaging, whilst a 30–40% dose reduction may be appropriate for soft tissue imaging (obtained by averaging dose reductions across all soft tissues). These suggested dose reductions are supported by the sufficient comparability of the bone, lung and soft tissue image quality between the reduced-dose Sn100kV images and the full-dose CRS images in Fig. 9. The equations for the CNR vs. ED linear fits specific to tissue type provided in supplementary data Appendix I, can be used to estimate the dose reductions which can be made with use of Sn100kV, in providing comparable image quality to CRS non-tin filter images.

Whilst two studies on the use of tin filter with PET-CT have previously demonstrated that ultra-low whole-body CT doses in the region of 0.1 mSv can be used for PET AC without significantly impacting PET quantification [23, 24], this is the first study to focus on the dose reduction for L/C level CT in PET-CT, and the results should also be valid for L/C CT in SPECT-CT, provided that the same tube voltages are offered. The large dose savings demonstrated in this study are similar to those observed in tin filter studies for standalone CT for bone and lung [10–15].

### Explanation of results

CNR is a widely accepted metric for evaluating image quality in CT. It is well understood that, in removing a greater proportion of lower energy photons from the X-ray beam, the tin filter provides a higher average beam energy as compared with standard filtration, if the same tube voltage is used. This in turn, is known to reduce image contrast in soft tissue and bone. With use of the tin filter at an even higher tube voltage of 140 kV, the average beam energy is considerably greater than that at 120 kV with standard filtration. Hence, the image contrast is considerably lower than the CRS scans in bone and soft tissue at Sn140kV (Fig. 5). Combined with a higher beam energy also imparting a greater absorbed dose, the noise reduction offered by the tin filter at Sn140kV is not sufficient to allow dose reduction for comparable CNR in bone and soft tissues (Fig. 5).

On the other hand, Fig. 5 shows that image contrast in bone and soft tissue is only slightly reduced at Sn100kV as compared with 120 kV with standard filtration, especially when bearing in mind that the HU scale starts at around − 1000. This phenomenon, combined with the tin filter removing such a large proportion of low energy X-rays from the beam, which are not useful to the image, means that noise is greatly reduced for the same dose, or dose can be greatly reduced for the same CNR.

When comparing Fig. 8a and b some differences can be seen in how much dose reduction each observer deems feasible with the tin filter for comparable image quality to standard filtration scans, with observer 1 generally accepting greater dose reductions than observer 2. Still, there is clear concordance between observers that considerable dose reductions are possible using the tin filter. The permissible dose savings for bone examinations were even greater than those for soft tissue for several reasons. Firstly, greater dose savings are indicated for imaging of higher contrast structures and abnormalities, since a given level of reduction in image contrast will be less noticeable when the image contrast is already very high, as compared with a relatively low-contrast structure. Secondly, soft tissue windows tend to have a narrower HU range than bone windows, hence a small absolute difference in HU when viewed in a soft tissue window will have a greater difference in greyscale appearance and thus image contrast. Lastly, ROIs were placed in multiple soft tissues, hence there is a wider range of dose reductions seen for soft tissue, as compared with bone which was represented by just one ROI. That said, the suggested dose-savings for soft tissue examinations were still large, albeit not as large as for bone.

### Clinical implementation

The data from this study suggests that CT dose reductions in the region of 30–40% and 85% can be made for soft tissue and bone examinations, respectively, for comparable L/C CT image quality to CRS protocols with standard filtration. On this basis, an 85% dose reduction has been implemented in clinical practice for L/C CT in NaF PET-CT with use of the Sn100kV/50 mAs setting in this department, given the excellent bone image quality at such marked dose reduction. For implementation of the suggested dose reductions in soft tissue imaging, such as for FDG PET-CT, the authors feel that a more careful clinical validation is necessary to compare visualisation of lymph nodes and metastases between dose-reduced tin filter and standard filtration scans.

Previous studies have shown that down to 0.1–0.2 mSv whole-body CT dose in a standard-sized patient or phantom (Sn100kV with 25–50 mAs) the impact on PET AC is negligible [23, 24]. If implementing an 85% dose reduction for bone examinations, the soft tissue CT visualisation in the bone scan may be inferior to the CRS protocol, since such an aggressive dose reduction is not indicated for soft tissue. However, for optimisation of CT image quality and radiation dose, the clinical purpose of the CT scan should be borne in mind. For example, in the case of bone imaging with ^18^F-NaF PET-CT at this Nuclear Medicine department in which an 85% CT dose reduction has been made with the tin filter, visualisation of bone and not lymph nodes, is the clinical purpose of the CT scan.

The findings in this study describe only the CT dose-savings possible with use of the tin filter. However, the PET-CT systems offering tin filter technology are also equipped with a state-of-the-art Stellar detector, which reduces noise thereby allowing dose reductions of 27-70% depending on tissue type [25], as well as large dose reductions offered by the second and third generation iterative reconstructions [26–28]. Even prior to implementation of the tin filter, this department’s 120 kV/20 mAs CRS L/C CT scan for ^18^F-NaF bone PET-CT examinations (1.4 mSv in a standard-sized patient), was already less than half of the reported Nordic median dose [4]. With the addition of the tin filter reducing this dose to just 0.2 mSv in a standard-sized patient, this department is delivering a CT dose for ^18^F-NaF PET-CT at a greater than 90% reduction compared with the Nordic median dose.

These combined state-of the-art CT technologies allow such low CT radiation doses in PET-CT, that in addition to reducing the supposed risk of inducing a fatal cancer in later life for patients already undergoing PET-CT, these greatly reduced CT doses can also potentially change molecular imaging practice. For example, we may see a greater transition from planar gamma camera to cross-sectional hybrid imaging, since lesion detection (sensitivity) is greater [29, 30] and the lower radiation dose is more easily justifiable. Furthermore, PET-CT may be more justifiable for younger populations and pregnant patients [31], instead of referrers being persuaded to choose lower-dose alternatives.

### Study design

This study examined CT dose reductions possible with the tin filter for L/C level CT image quality in PET-CT, compared with the CRS. For simplicity, all protocols used CARE kV in ‘semi’ mode, meaning that a fixed CT tube voltage was used, rather than utilising the full CARE kV functionality to optimise tube voltage specific to patient/phantom size, as is performed clinically in this department. Previous phantom acquisitions (data not shown) suggest that 100 kV is deemed optimal by the CARE kV application for the standard phantom configuration, and 120 kV for the obese configuration. Whilst tube voltage optimisation with CARE kV for the CRS scan would have resulted in no dose differences for the obese phantom, it may have reduced dose in the standard-sized phantom, with a previous study demonstrating up to 9.3% dose reduction with an optimal tube voltage of 100 kV in phantoms [32]. However, even assuming the full 9.3% dose reduction could be realised in the CRS scan through tube voltage optimisation at 100 kV, this would have contributed little difference to the observed relative dose savings with the tin filter (around 1% difference for bone and 7% for soft tissue).

The methods section details that scan settings were optimised for bone image quality, with the CARE kV sliding bar setting set to the bone exam type, and choice of the spine organ characteristic for tube current modulation, since this department was most interested in implementing the tin filter for ^18^F-NaF bone PET-CT from the outset. However, these settings would have no impact on the relative dose savings between tin filter and CRS scans with standard filtration, or the absolute dose required to achieve a given level of image quality, in any tissue type.

This study reported relative dose savings which could be made with the tin filter as compared with standard filtration protocols for comparable image quality, and the absolute effective doses which would have been delivered to a patient, by use of a whole-body multiplication factor to convert DLP to ED in standard-sized patients, as described by Inoue et al. [21]. AAPM Report 204 describes the influence of patient size on absorbed dose, with smaller patients incurring a greater absorbed dose and larger patients incurring a lower absorbed dose, for a given scanner output. This can lead to absorbed doses and thus effective doses being underestimated in smaller patients and overestimated in larger patients. To remedy this, SSDE conversion factors were published in AAPM Report 204, to allow normalisation of absorbed dose values for patient size. Yet, the dose values presented in this study had not been normalised for size, since the published conversion factors are applicable only to the thorax-abdomen-pelvis scan range and not for the vertex-to-knee scan range used in this study. This phenomenon has no impact on the relative dose reductions achieved with tin filter protocols as compared with standard filtration protocols for comparable image quality. However, it should be borne in mind that the absolute effective doses quoted for the obese configuration may be overestimated in this study, and the absolute doses for the standard phantom may be slightly underestimated.

A ROI placed in fat was used in this study as a reference for measuring contrast. In this phantom, this is placed in an area which in a real patient may comprise a mixture of fat and muscle. Hence, if calculating CNR in a patient, a different reference region may be required.

This investigation has not addressed the potential impact on PET images, when using a CT scan for PET AC acquired using the tin filter at the investigated settings. However, previous work has demonstrated that with Sn100kV at the mAs settings advocated for in this study (minimum 50 mAs), there is a < 2% difference in PET quantification at soft tissue and bone equivalent densities [23].

### Limitations and future work

This study evaluated image quality in a phantom with normal morphology, without representation of abnormalities typically seen in clinical PET-CT examinations. Imaging of the PBU-60 phantom is an adequate and ethical means to assess differences in dose and image quality in the human body when comparing different CT exposure settings. Nevertheless, a patient-based study validating the clinical adequacy of real patient images exhibiting adequate visualisation of real clinical abnormalities would provide additional reassurance for implementing these dose reductions with use of the tin filter in clinical practice.

In this study, the doses and image quality provided by the tin filter were investigated at the two available tube voltages: Sn100kV and Sn140kV. However, other CT systems may have other tin filter tube voltages available. This study has demonstrated that the dose and image quality delivered by the tin filter is highly dependent on the tube voltage, and thus, further studies should be undertaken to evaluate the dose reductions which can be made with the tin filter at the other available tube voltages.

This study only compared dose savings with tin filter for comparable CNR and visual interpretation but did not evaluate differences in artefacts between tin filter and standard filtration scans, which should be evaluated in future work. Lastly, this study only compared dose savings with the tin filter in adult body sizes, and future work should also evaluate the possible dose savings with the tin filter in paediatrics.

## Conclusions

This study has demonstrated that the CT tin filter used at Sn100kV allows large CT dose savings for L/C purposes in adult PET-CT examinations, with dose reductions in the region of 85% for bone and 30–40% for soft tissue. Such large dose reductions seen with the tin filter, as well as other state-of-the-art CT dose reduction technologies, does allow L/C level CT to be performed at genuinely ‘low dose’ or even ‘ultra-low-dose’ depending on the clinical application. Such great magnitudes of CT dose reduction in PET-CT, in addition to reducing the risk of inducing a fatal cancer in later life, also has the potential to optimise how CT is practiced in molecular imaging.

## Electronic supplementary material

Below is the link to the electronic supplementary material.

Supplementary Material 1

## Data Availability

The datasets used and/or analysed during the current study are available from the corresponding author on reasonable request.

## Abbreviations

^18^F-FDG: Fluorine-18 labelled fluorodeoxyglucose
^18^F-NaF: Fluorine-18 labelled sodium-fluoride
AAPM: American Association of Physicists in Medicine
AC: Attenuation correction
ADMIRE: Advanced modelled iterative reconstruction
Br38: Regular body kernel with resolution index 38 for soft tissue CT reading
Br51: Regular body kernel with resolution index 51 for low-dose bone CT reading
CNR: Contrast-to-noise ratio
CRS: Clinical reference standard
CT: Computed tomography
DLP: Dose length product
ED: Effective dose
HU: Hounsfield units
kV: kilovolt
L/C: Localisation/characterisation
mA: milliamperes
mAs: milliampere seconds
mAs-ref: Reference mAs
mGy.cm: milligray centimetres
mSv: Millisievert
PET: Positron emission tomography
ROI: Region of interest
SD: Standard deviation
Sn: Symbol for the chemical element tin
Sn100kV: Tin filter applied to X-rays generated using a peak tube voltage of 100 kV
Sn140kV: Tin filter applied to X-rays generated using a peak tube voltage of 140 kV
SPECT: Single photon emission computed tomography
SSDE: Size-specific dose estimate
