# Supplementary material for: CT radiation dose reduction with tin filter for localisation/characterisation level image quality in PET-CT: a phantom study
Source: EJNMMI Phys. 2024 Nov 25;11:100. doi: 10.1186/s40658-024-00703-6 (PMC11589033; doi:10.1186/s40658-024-00703-6)
Supplement: Supplementary file 1 — Supplementary Material 1 [file 40658_2024_703_MOESM1_ESM.docx]

Appendix I: equations for linear fits for CNR versus ED with Sn100kV

| **Phantom configuration** | **Body region** | **Linear fit equation**  where x = ED and  y = CNR relative to fat | **R^2^ value** |
| --- | --- | --- | --- |
| Standard | Brain | y = 2.07x + 1.11 | 0.940 |
|  | Lung | y = 32.11x + 32.02 | 0.971 |
|  | Bone | y = 10.77x + 8.02 | 0.916 |
|  | Liver | y = 3.26x + 1.31 | 0.918 |
|  | Kidney | y = 0.90x + 0.41 | 0.862 |
| Obese | Brain | y = 0.96x + 1.35 | 0.966 |
|  | Lung | y = 14.76x + 30.33 | 0.938 |
|  | Bone | y = 6.23x + 4.95 | 0.973 |
|  | Liver | y = 0.93x + 1.01 | 0.849 |
|  | Kidney | y = 0.33x + 0.52 | 0.801 |

CNR = contrast-to-noise ratio; ED = effective dose
